# Size and Velocity Distribution of Negatively Charged Helium Nanodroplets

**DOI:** 10.1021/acs.jpca.1c05619

**Published:** 2021-08-27

**Authors:** F. Laimer, F. Zappa, P. Scheier

**Affiliations:** † Institut für Ionenphysik und Angewandte Physik, Universität Innsbruck, Technikerstr. 25, A-6020 Innsbruck, Austria; ‡ Departamento de Física-ICE, Universidade Federal de Juiz de Fora, Campus Universitário, 36036-900 Juiz de Fora, Minas Gerais, Brazil

## Abstract

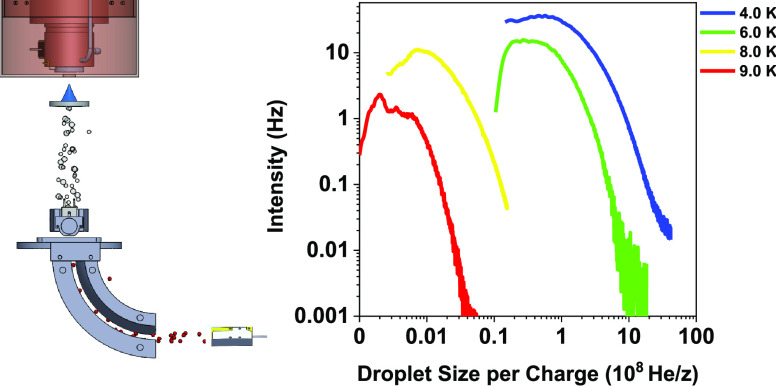

Precharged helium nanodroplets can be used in doping experiments with the advantage that they are amenable to size selection with electrostatic fields, therefore adding a useful tuning parameter for dopant growth. For all these applications, the knowledge of the size distribution of charged droplets is an essential parameter, which we have so far assumed would be equivalent to that of their neutral precursors. Here, this assumption is experimentally investigated for negatively charged clusters for temperatures between 4 and 9 K at a stagnation pressure of 2 MPa. We observe a dependency of the velocity of the droplets on mass per charge, especially at the lowest temperatures of the investigated range, and values 20% lower than those known from the literature. Below 6 K, a large deviation from the literature is also found for the average droplet sizes. This information has to be taken into consideration in future experiments where large, charged droplets are sought to produce large dopant clusters. Possible origins for this deviation are discussed in the text.

## Introduction

Helium nanodroplets (HNDs) have been studied since the pioneering experiments from Becker and co-workers in 1961.^1^ In subsequent experiments, the groups of Gspann,^2−4^ Toennies,^5−8^ and Northby^9−11^ investigated the ionization of HNDs. Recently, efficient formation of He-tagged ions was also demonstrated for doped HNDs^12^ that provide perfect targets for messenger-type spectroscopy.^13−17^ Droplets containing up to 10^11^ He atoms have been used to form nanoparticles and nanowires grown along quantized vortices.^18−21^

Quite recently, we demonstrated that multiply charged, pristine HNDs can also be efficiently doped to form ultracold atomic and molecular cluster ion beams,^22^ with various applications so far.^23−28^ By bending the charged helium droplet beam before doping, the setup allowed for a precise control of pickup statistics and dopant cluster sizes. The pickup and ionization cross-section of a helium droplet (containing *n* He atoms) scales with the droplet’s surface and thus with *n*^2/3^. The typical log-normal size distribution of neutral HNDs together with a Poisson pickup statistic results in a log-normal distribution of dopant clusters^22,29^ or nanoparticles.^30,31^

The average size of HNDs was first determined in the early 80s by the group of Gspann based on a combined acceleration and time of flight (TOF) method.^2−4^ They determined average droplet sizes between 10^5^ and 10^7^ atoms. About a decade later, Northby and co-workers measured size distributions of positively and negatively charged HNDs by retarding field analysis^10^ and deflection in an electrostatic field orthogonal to the droplet beam.^9^ Henne and Toennies improved the deflection method and determined droplet size distributions for negatively charged droplets up to an average size of 10^8^ He atoms.^5^ Knuth and Henne compared distributions of negatively and positively charged droplets.^32^ Fárník et al. observed a strong dependence of the size distributions of HNDs on the ionization parameters, that is, electron energy and electron current.^6^ Very recently, Laimer et al. directly determined the appearance size for doubly charged HNDs to be 10^5^ He for cations^33^ and 4 × 10^6^ for anions.^34^

The group of Vilesov developed a titration method to determine the average size of micrometer-sized HNDs containing 10^5^ to more than 10^11^ He atoms.^35^ In a later study, they determined the size of a single HND via the number of ejected He_2_^+^ ions when subjected to multiple ionization by electron impact.^36^ Both methods can be applied for the largest droplet sizes and for pulsed nozzles too. Finally, microscope images have also been used to estimate the size of micrometer-sized HNDs.^37,38^ All experiments mentioned above were performed with continuous flow nozzles with diameters typically of a few micrometers.

Pulsed nozzles recently became quite popular^39−46^ as they provide higher peak flux and larger droplets, and they are less demanding on the pumping speed. However, the average droplet size changes within every pulse, and the droplet beam is less stable. In our opinion, the deflection method is the most direct way to select specific nanodroplet mass per charge and because a constant and narrow velocity distribution of the HND beams is essential, continuously operated HND sources are our preferred choice.

In experiments with droplets that are selected on their mass per charge, we repeatedly tried to verify the data available in the literature for neutral HND sizes; however, surprisingly, the preliminary results always indicated a substantial discrepancy below 7 K, even for negatively charged HNDs, where multiple charging of the droplets is expected to be less prevalent. This observation prompted us to study the size distribution and velocity of negatively charged HNDs in detail. The present study reports on these efforts. Here, charged droplets are obtained via electron attachment to neutral droplets, produced in a supersonic expansion of precooled helium at 2 MPa, for temperatures ranging between 4 and 9 K. We used two different electrostatic energy analyzers to obtain TOF and ion intensity distributions as functions of the applied voltage difference between analyzer plates. The TOFs can be converted to droplet velocities and, when combined with analyzer voltages, used to obtain velocity and intensity distributions as a function of mass per charge. A strong dependence of droplet velocity on mass per charge is observed, especially in the lower temperature range of our measurements. Average-sized droplets at each temperature present velocities 20% lower than those in the literature, where different droplet masses are not generally discriminated in velocity measurements. The average sizes obtained here seem consistent with the previously published data on neutral droplets down to temperatures between 6 and 7 K, but below this point, saturation is evident even at the lowest practical electron currents of our experiment. The data presented here will be relevant for the planning of any experiments with charged helium droplets where the droplet size per charge is the most important parameter in the optimization of dopant pickup and mass selection.

## Experimental Methods

**Figure 1:**
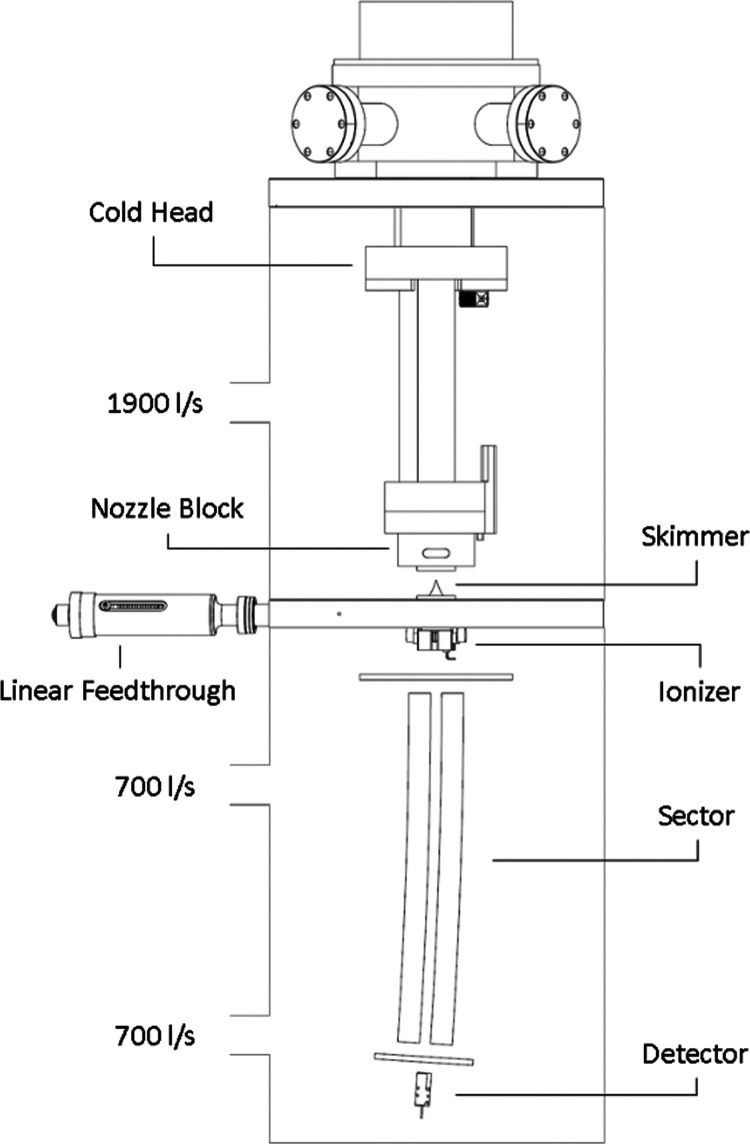
Schematic drawing of the experimental setup incorporating the 5 m radius cylindrical energy analyzer. Without helium flow, both chambers have a base pressure of 10^–6^ Pa. During operation at 4 K, the upper chamber resides at a pressure of 10^–2^ Pa, while the bottom chamber reaches up to 10^–4^ Pa.

A schematic drawing of the setup is shown in Figure 1. Neutral HNDs are produced by the expansion of helium gas (Messer, 99.9999% purity) into vacuum through a continuously operated nozzle (Lenox Laser SS-2-VCR-2-VS-5) with a 5.4 μm opening (see the Supporting Information) under a stagnation pressure of 2 MPa. The nozzle was characterized using a scanning electron microscope and installed on an oxygen-free copper cylinder mounted onto the cold finger of a liquid helium cryocooler (SHI RDK-408D2/RP-182B2S). Temperatures between 3.8 and 30 K can be set via a PID-controlled heating system directly attached to the front of the nozzle block (Lakeshore 325, Lakeshore DT-670-CU, Ohmite 825). The temperature of the nozzle largely determines the formation process and the resulting size distribution of the droplets.^35^

After passing a 0.5 mm skimmer (Beam Dynamics Ni), the droplets are ionized by electron impact. To guarantee maximum overlap of the neutral droplet beam and the electron beam, the ionizer is mounted onto a movable sled which can be positioned relative to the skimmer by a bellow-sealed mechanical linear feedthrough (Hositrad 2″ LF). The ionizer itself is based on an old Nier-type electron impact design.^22^ In this configuration, the interaction between electrons and the helium beam occurs inside a partially closed Faraday cage, usually called the “ion block.” The electron energy is established as the potential difference between the emission filament and ion block, which was held at ground potential. Electron energy resolution in this configuration is mainly limited by the voltage drop across the emitting filament and is typically between 1 and 2 eV. For TOF measurements, the electron beam is pulsed by modulating the electron energy with a high-speed voltage switch (Behlke HTS 41-06-GSM, Siglent SDG 1032X, 1 ns rise time, 20 μs pulse width). The electron beam width in the interaction volume between the electrons and the helium droplets is estimated to be less than 1 mm, which is based on the aperture for the electron beam in the ion block.

Depending on the pressure and temperature of the expanding liquid, spraying, jet branching, and flashing have been observed.^38^ Particularly, at temperatures below 4 K, the divergence of the beam exceeds 5°. With an acceptance angle of 2° (0.5 mm skimmer opening at 13 mm distance to the nozzle), our skimmer is expected to exclude side jets and parts of the main droplet beam at low temperatures. The apertures in the ion block are assumed to be sufficiently wide to allow the passage of this diverging beam without impediment.

To decrease the influence of multiple charging on the size distribution, both the possibility of multiple electron hits per droplet and the formation of two charges by one incident electron have to be kept low. This can be achieved by low electron currents and electron energies below 46 eV.^47^ Therefore, we operated the electron ionizer at an electron energy of 22 eV to promote the formation of anions. The electron current was reduced to 50 nA, the lowest current that could be achieved while still maintaining constant emission from the filament. Any further current increase leads to a pronounced compression of the size distributions and overall signal increase.

After ionization, the droplets were separated for their kinetic energy by either one of the two different electrostatic energy analyzers (90° spherical, 0.07 m radius, 0.02 m electrode distance; 3.67° cylindrical, 5 m radius, 0.01 m electrode distance). The total flight distance between ionization and detection was calculated from the technical drawings of each analyzer to be 330 and 645 mm, respectively. In both cases, the ions were detected with a continuous electron multiplier (Dr. Sjuts Optotechnik KBL 510, Winkelnkemper Engineering PAD 06DS). For positively charged droplets, detection with an electron multiplier is possible because the ionization energy of helium is high enough to produce a free electron upon collision with the detector surface even at very slow velocities. Although less obvious, the same holds true for negative clusters because the charge carrier is most likely a He^*–^ anion.

Given an applied electric potential difference *U* between the deflection plates of the energy analyzer, the droplet size *n* can be calculated using the following expression:
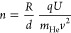
1where *q* is the droplet charge*, R* is the analyzer radius, *v* is the droplet velocity*, m*_He_ is the mass of a ^4^He atom, and *d* is the distance between the plates. Care was taken so that the voltage of each deflection plate for a given potential difference results in a ground potential for the central beamline path so that the velocity of each charged droplet that exits the analyzer does not change appreciably during its flight. This was confirmed via computer simulations for the trajectories of the droplets inside the devices (see the Supporting Information).

It is well known that expansion pressure and temperature are defining parameters for the beam velocities in supersonic helium expansion. Also, a size-dependent droplet velocity has been observed previously by Buchenau^48^ and Henne.^49^ To determine the size-dependent velocity of the droplets produced in our experiment, for each temperature, we obtained TOF measurements for various values of the deflection voltage *U*, spanning the whole range where the ion signal was significant. The arrival times of the droplets at the detector were resolved with a multichannel analyzer card (FastcomTec P7888-1E).

## Results and Discussion

**Figure 2:**
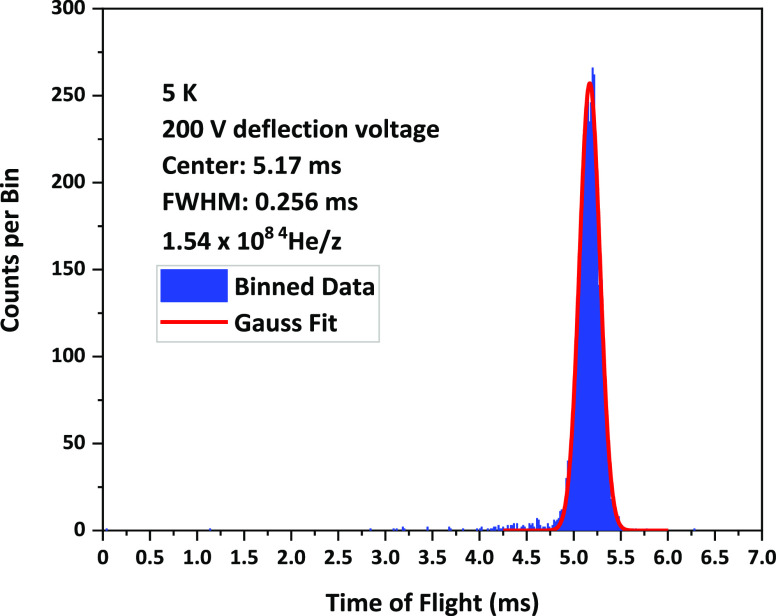
TOF measurement of size-selected droplets at 200 V deflection voltage at 5 K nozzle temperature. To determine the velocity for the size-selected droplets, a Gaussian profile (red curve) was fitted to the measured data (blue curve). Using expression 1, a mass per charge ratio of 1.54 × 10^8 4^He/z is calculated for the given deflection voltage and velocity. The droplets were ionized by electron impact with a kinetic electron energy of 22 eV and an electron current of 50 nA.

An example of a typical TOF measurement for a voltage difference of 200 V between the plates of our 5 m radius analyzer and the cryostat operating at 5 K is presented in Figure 2. Each time bin of 16,384 ns corresponds roughly to the electron pulse duration and is practically negligible compared to the total flight time. In agreement with previous measurements for anionic droplets,^49^ only a single peak could be observed. From the Gaussian fit of the peak and the calculated flight path of each analyzer, one can obtain the average velocity and speed ratio of the transmitted droplets as a function of analyzer voltage with an uncertainty lower than 1%. The peak width of only 5% of the center value validates, in our opinion, the use of the energy analyzer as a mass per charge selector.

**Figure 3:**
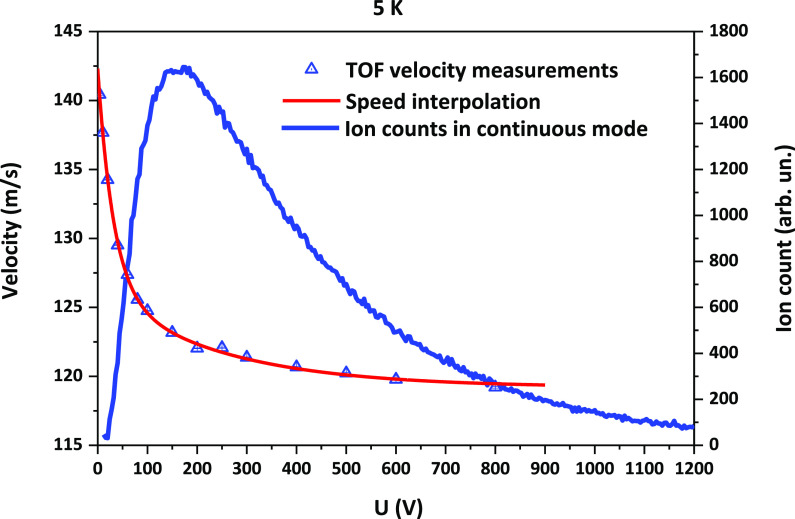
Up triangles: centroid velocities of the TOF peaks for each voltage difference between analyzer plates *U* (V); continuous red line: exponential fit to experimental velocity values, used merely for interpolation; blue line: ion intensity when the electron gun is operated in continuous mode.

We present in Figure 3 the results obtained at 5 K for the centroid velocity of the transmitted ions as a function of analyzer voltage. Given the high duty cycle of the TOF experiments, we chose to operate the electron gun continuously, without pulsing, when measuring the size distributions, which results in almost a continuous curve of ion intensity versus analyzer voltage, as also presented in Figure 3.

**Figure 4:**
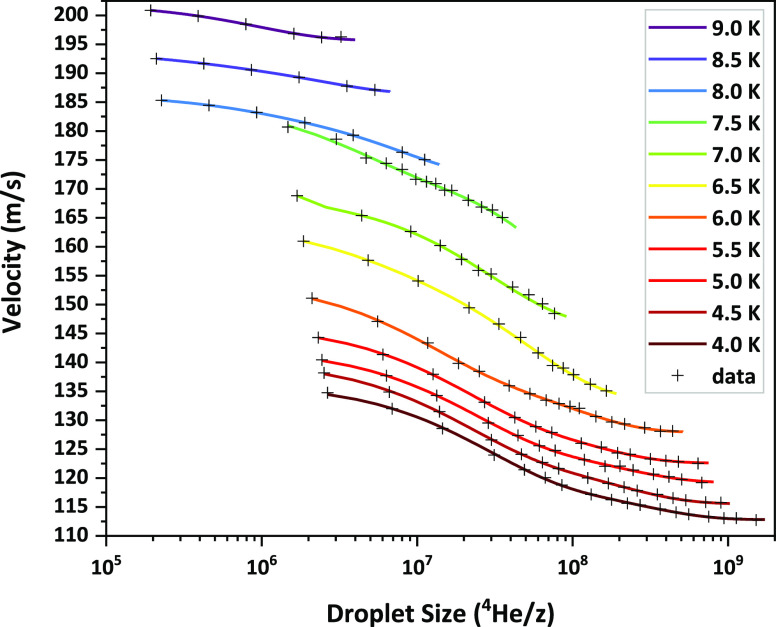
Measured droplet velocities (black crosses) for anionic size-selected droplets are shown for a nozzle temperature range from 4 to 9 K at 2 MPa expansion pressure. The solid, colored lines are shown as a guide to the eye, as well as for obtaining interpolated values. Ionization was carried out by electron impact with an electron current of 50 nA at an electron energy of 22 eV.

Using expression 1, we can assign the velocity at a given analyzer voltage to a specific mass per charge ratio. In the case of Figure 2, in particular, one obtains 1.54 × 10^8^ helium atoms per charge. Following this procedure, we obtain velocity for helium droplets as a function of mass per charge for temperatures ranging between 4 and 7.5 K in the case of the large analyzer (5 m radius) and between 8 and 9 K in the case of the small analyzer (0.07 m radius). The results are presented in Figure 4.

Ion intensity curves as a function of analyzer voltage, similar to the one depicted in Figure 3, can be converted to ion intensity curves as a function of the cluster size using expression 1 and the velocity interpolated from Figure 3. Care has to be taken to compensate for the change in the absolute resolution of the electrostatic deflector with increasing deflection voltage. This implies dividing the ion yield by the analyzer voltage at each point, as discussed in the Supporting Information. The resulting normalized ion yield plotted as a function of the droplet size can be fitted with a log-normal curve, as has been observed in previous deflection experiments^5,6^ and as depicted in Figure S8 of the Supporting Information. The average values of these size distributions represented by the center value of the log-normal fit are compared with those in the literature^35,49,50^ and are plotted in Figure 6, as well as listed in the Supporting Information. Other measurements reported in the literature have very different experimental conditions and are not included in the graph.^51,52^

### Comparison to the Literature for Different Temperatures

**Figure 5:**
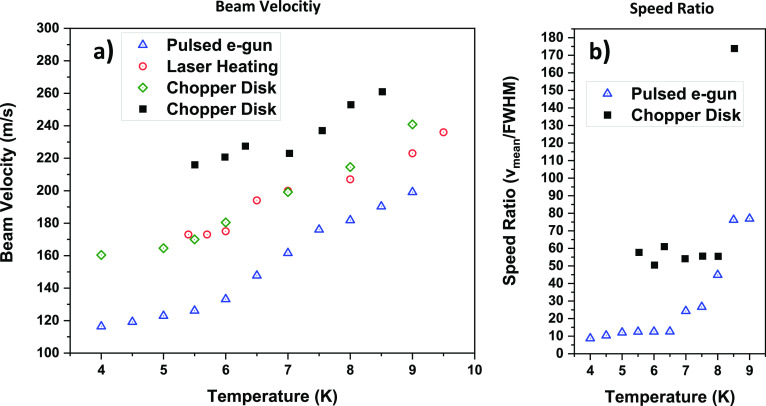
(a) Beam velocities at the mean droplet size as a function of nozzle temperature (blue triangles) measured by pulsing the electron acceleration voltage of the cross-beam ionizer are compared to pulsed laser heating measurements by Vilesov^35^ (red circles) and random chopper disk measurements by Henne^49^ (green diamonds) and Buchenau^48^ (black squares). (b) Speed ratios as a function of nozzle temperature are shown as blue triangles, which are calculated by dividing the droplet velocity of the mean droplet size by the velocity difference between both ends of the full width at half maximum (FWHM) of the droplet size distribution. Solid squares again represent data by Buchenau.^48^

**Figure 6:**
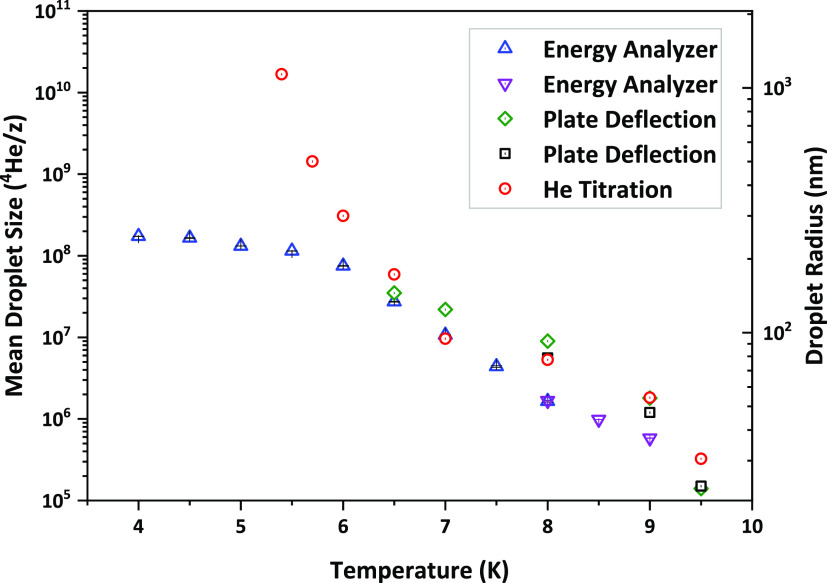
Mean droplet sizes and radii as a function of nozzle temperature. Up triangles are data obtained using the 5 m radius energy analyzer at 2 MPa expansion pressure for anionic droplets produced with 22 eV kinetic electron energy and 50 nA electron current. Down triangles mark data obtained with the 0.07 m radius analyzer for the same conditions. Mean droplet sizes obtained through deflection by electrostatic parallel plates by Henne^49^ and Samelin^50^ are depicted as green diamonds and black squares, respectively. Neutral mean droplet sizes determined through He-titration by Vilesov^35^ are shown as red circles.

As can be seen in Figure 4, for certain temperatures, the measured velocities span a range much wider than the single peak widths of the individual measurements. This makes the comparison with data reported in the literature, which are in most cases insensitive to the droplet size, not so straightforward. For any given temperature, the intensity of charged droplets as a function of mass per charge follows very well a log-normal distribution. Therefore, we will define the single mean velocity value for the supersonic expansion at each temperature as being the velocity for the average-sized droplet at that temperature, as taken from Figure 6, interpolated from the data of Figure 4. The results of this procedure can be seen in Figure 5a). Despite the general behavior agreeing well, our velocity values are 20 to 25% lower overall. Given the size-dependent nature of our measurements, however, it is difficult to evaluate the significance of this discrepancy.

In a similar manner, defining a single speed ratio in a size-dependent velocity distribution can be ambiguous. In order to compare our results with the ones that do not discriminate for the cluster size, we shall define here the speed ratio for our data as the ratio between the velocity of the mean droplet size and the difference in the droplet velocity at each side of the half maximum of the fitted log-normal distribution of droplet sizes. We chose this definition because in experiments where all droplet sizes contribute, the result should be the convolution between the size distribution and the velocity distribution of each size. These speed ratios are shown as a function of nozzle temperature in Figure 5b. Speed ratios for helium droplet beams are not available in the literature, with the exception of the data by Buchenau.^48^ In this work, a similar trend was reported, where the speed ratio decreases sharply below a certain nozzle temperature, eventually leveling out at even lower temperatures. This can be explained by a change in expansion regimes as the temperature is lowered. Below a critical point, decreasing temperature leads to further condensation of gaseous helium, which increases the release of heat of condensation, leading, in turn, to a broadening of the velocity spread. At even lower temperatures, in the supercritical regime, the disintegration of liquid helium into HNDs is expected to balance the effect of condensation, and the velocity spread stays constant.

In Figure 6, we can see that the average sizes obtained in our experiment are arguably in good agreement with the previously reported values down to about 7 K. Below this point, a much more pronounced deviation from titration measurements by Vilesov^35^ can be seen. In order to interpret this result, first of all, we recall that in the titration measurements, the HNDs propagate through the experiment as neutral particles, and ionization is performed only at the very last step as a way to obtain the partial pressure increase that is produced by the droplet beam colliding with the chamber walls. Because the droplet beam is ionized before mass selection in our experiment, one should consider the possibility for droplets to be multiply charged, which would result in a lower apparent droplet size.

Assuming that the electron collision cross-section scales with the geometrical cross-section of the droplets and admitting the possibility that these droplets could be multiply charged, the probability that a droplet is singly charged would have to go down as the size of the neutral precursor increases. In fact, very recently, we have demonstrated the existence of multiply charged negative helium droplets^34^ with charges at least up to 5 and a critical size of only 4 million helium atoms for doubly charged species. One could interpret our present results as indirect evidence for the existence of droplets with hundreds of negative charges. This remarkable observation also has deep consequences for our experiments where we would like to control the doping of charged HNDs in order to obtain specific dopant cluster sizes. As it has already been shown,^22^ we have evidence that separate charge centers within HNDs can act as individual nucleation seeds, impacting the statistics of dopant cluster growth. Highly charged droplets will tend to produce smaller dopant clusters.

In addition to the multiple charging hypothesis, we also need to explore other possible explanations for the discrepancy of our values in comparison to the literature. For instance, at the electron energy used in this work, multiple electron collisions in the same droplet could also produce a mixture of excited He* and He^*–^ that can recombine to He^+^,^53^ effectively reducing the negative ion signal of droplets in respect to their geometric cross-sections. An attempt to overcome this possibility by choosing electron energies below the excitation threshold led to an insufficient ion signal. Another observation is that because of their large cross-sections, HNDs are known to readily pick up water molecules.^54^ Even though our background pressure is 10^–6^ Pa and very good care was taken to bake the chamber thoroughly to decrease the water partial pressure to a minimum, the possibility that very large clusters are contaminated cannot be ruled out for the sizes reported in this work. Taking the stated background pressure and the length of the flight path into account, droplets already containing 10^7^ He atoms are expected to have more than one collision with the background gas particles. The presence of doped droplets has a nontrivial effect on the detection efficiency of our apparatus. By increasing the water content of the residual gas on purpose, we have observed a detrimental effect on the detection efficiency in negatively charged helium droplets as a whole, but we could not observe a change in the shape of the distributions that could explain the discrepancy with the literature.

## Conclusions

Kinetic energies per unit charge and velocities were simultaneously determined for negatively charged helium nanodroplets obtained from a continuous supersonic expansion at stagnation temperatures between 4 and 9 K and a pressure of 2 MPa. From this data, it was possible to observe a dependence of the droplet velocity not only on stagnation temperature and pressure but also on the specific mass per charge of each droplet. Operating the electron gun in continuous mode, and with the information about the size-dependent velocities at different temperatures, we obtained new data for the droplet size probability distribution functions up to mass per charge values equivalent to 10^9^ helium atoms. These distributions were fitted with log-normal functions, and the average droplet sizes are presented as a function of temperature.

The main motivation of the work was initially to verify the validity for the case of negatively charged droplets, in this range, of the data available in the literature for neutral HNDs. We have obtained good agreement between our measurements of mean droplet sizes and previous measurements in the literature down to a stagnation temperature of 7 K. Below this temperature, several effects may play a role in producing results that are progressively more discrepant to measurements on neutral droplets. The most plausible cause for the discrepancy in our view is the presence of multiply charged droplets with up to hundreds of negative charges, reducing their effective mass per charge. Given the evidence that charge centers can act as independent nucleation sites, this would have the implication that the size of dopant clusters grown in charged HNDs could be limited by the charging process and not only by the geometrical pickup cross-section even for negative droplets.

## Supporting Information Available

Brief description of computer simulations performed in SIMION; detailed description of data analysis; droplet sizes compiled in the tabulated form; and scanning electron microscopy image of the nozzle. (PDF)

## Abbreviations

HNDs: Helium nanodroplets
TOF: Time of Flight.
