# Supplementary material for: Size and Velocity Distribution of Negatively Charged Helium Nanodroplets
Source: J Phys Chem A. 2021 Aug 27;125(35):7662–9. doi: 10.1021/acs.jpca.1c05619 (PMC9282675; doi:10.1021/acs.jpca.1c05619)
Supplement: Supplementary file 1 — jp1c05619_si_001.pdf [file jp1c05619_si_001.pdf]

Size and Velocity Distribution of Negatively Charged Helium Nanodroplets

F. Laimer[1], F. Zappa[1,2] and P. Scheier[1]*

[1] Institut für Ionenphysik und Angewandte Physik, Universität Innsbruck, Technikerstr. 25, A-6020, Innsbruck, Austria

[2] Departamento de Física-ICE, Universidade Federal de Juiz de Fora, Campus Universitário, 36036-900, Juiz de Fora, MG, Brazil

A) Computer simulations

In order to access the behavior of our energy analyzers we transferred the CAD drawings into the simulation software SIMION (as shown in Figure S1) and performed a series of tests that mimic the experimental runs.

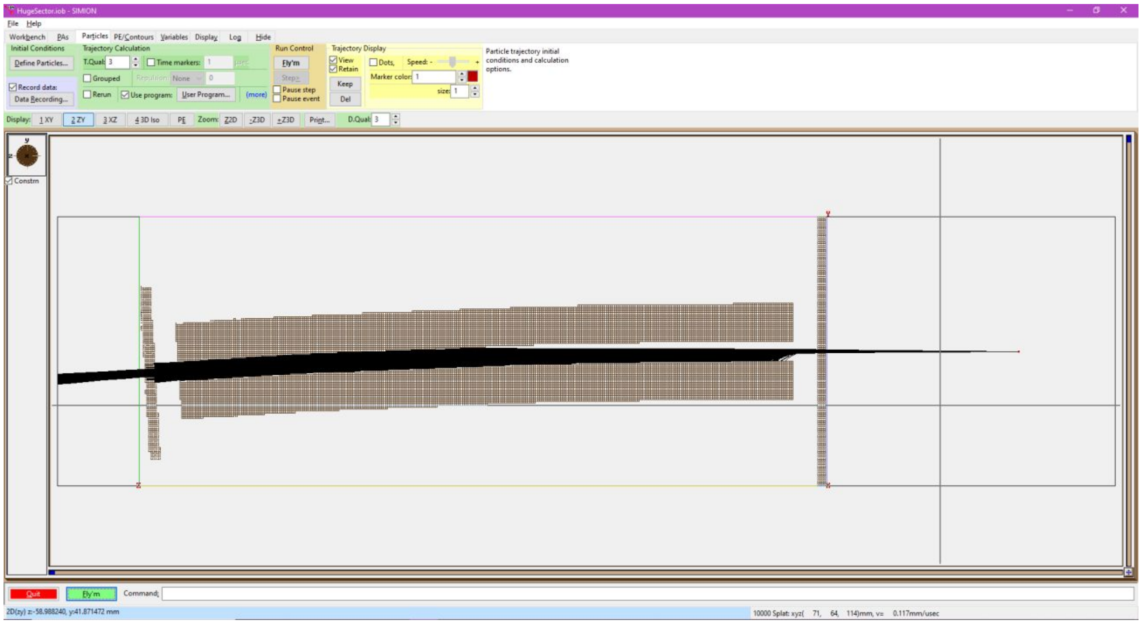

Figure S1. Screenshot of the simulation environment in SIMION for the spherical energy analyzer with 5 m radius. The origin of the particles coincides with the nozzle position in our experiment. The initial velocities of the particles were sampled inside a solid cone with the same acceptance angle of the nozzle-skimmer geometry. Different simulations were performed where the moduli of the velocity vectors were sampled over gaussian distributions of different center values and a tentative spread of 10%.

The first test was to verify the validity of the assumption that the potential surfaces between the analyzer plates can be approximated by the solution for two concentric spheres. We would like to assume that the principal trajectory of the ions through the analyzer coincides with the center line between the plates and that the potential there is zero. This is important in order to guarantee that the ions maintain a constant speed throughout the whole trajectory. For two concentric spheres, with the condition that the potential is zero in the middle, it is a textbook exercise to prove that the outer plate potential, V_2, is a function of the inner plate potential V_1 and the radii of the spheres, through the expression

$$V_2 = -V_1 \frac{R_1}{R_2} \tag{1}$$

where R_1 is the radius of the inner plate and R_2 is the radius of the outer one. In the case of the 5 m radius analyzer, the plates have practically symmetrical voltages. We verified that the potential is essentially zero at the center of the plates.

Various log-normal distributions for particle mass were used to sample initial conditions of the simulated ion trajectories inside the energy analyzers. One example of such a distribution can be found in Figure S2.

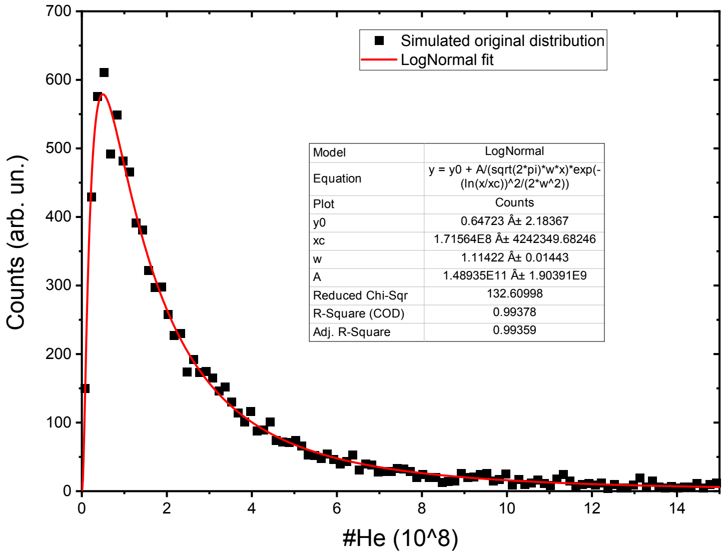

Figure S2. Example of log-normal distribution of particle mass used to sample ions for subsequent simulations. In this case the center of the distribution was aimed to be 1.7×10^{8} helium atoms.

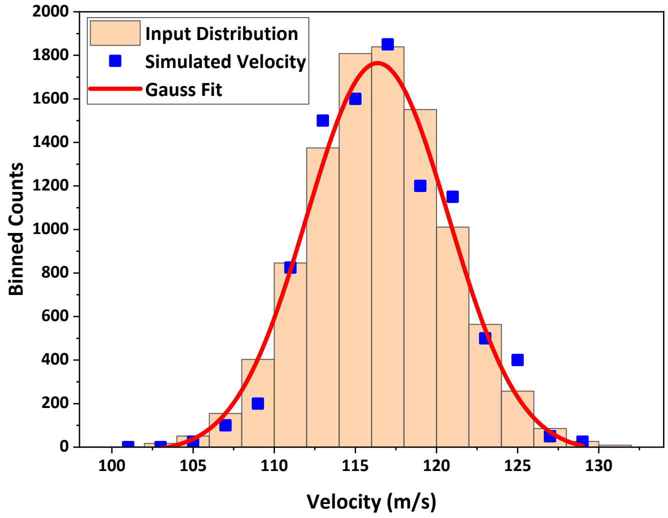

Figure S3. Computer simulation (SIMION 9) for helium droplets with a lognormal size distribution centered at x_c = 10^{8} helium atoms and a speed of 116 m/s with Gaussian speed distribution, flying through the cylindrical electric analyzer. Plate voltages in this case are symmetrical ± 100 V. The simulation was used to verify the influence of the analyzer plate voltages on the time flight and on the distribution of transmitted ions. The distribution of the simulated droplet velocities after passing the analyzer (blue squares) show no significant deviation from the starting distribution (orange bars), as can be seen by the Gaussian profile (red) fitted to the simulated velocity.

By recording the time of flight of the particles arriving at the virtual detector and converting the corresponding distribution to velocity we found out that the resulting distribution is indistinguishable from

the originally sampled speed distribution for the particle´s initial conditions, as can be seen in Figure S3. We conclude that the assumption of a constant speed for each particle is verified.

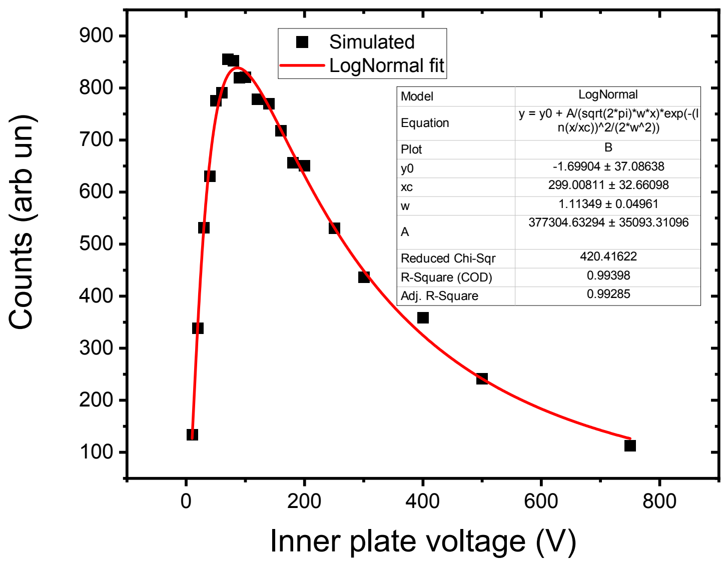

Figure S4. Simulated ion signal at the detector position as a function of the inner plate voltage, using the log-normal mass distribution of Figure S2.

Using the fact that the velocity of the particles remains unchanged during the trajectory, one can relate the total voltage difference between the analyzer plates and the number of helium particles per unit charge in a droplet by the expression

$$N = n/i = \frac{R}{d}\frac{eU}{m_{He}v^2}\#(2)$$

where i is the charge state and e is the elemental charge. By simply applying this conversion factor to the curve of Figure S4 we obtain the curve depicted in Figure S5.

It is clear that the distributions of Figures S2 and S5 do not match. We found the explanation for this effect in reference 1 of this supplement. In practice, because of the finite acceptance spread of different trajectory radii and the natural spread of velocities, we can think of the number of helium atoms per unit charge of a given cluster as being a function of two parameters

$$N(k,U) = k\,U\#(3)$$

and the signal recorded at the detector can be written in the form

$$I(U) \approx f(k)\Delta k\#(4)$$

where $f(k)$ is the probability distribution of particles having different trajectory radii and velocities within the acceptance range Δk consistent with the voltage U. The Jacobian transformation between $f(k,V)$ and $f(N)$, $\left|\frac{\partial N}{\partial k}\right|$ is equal U. Therefore, the true distribution of the number of Helium atoms per charge in a cluster is given by

$$f(N) \propto I(U)/U\#(5)$$

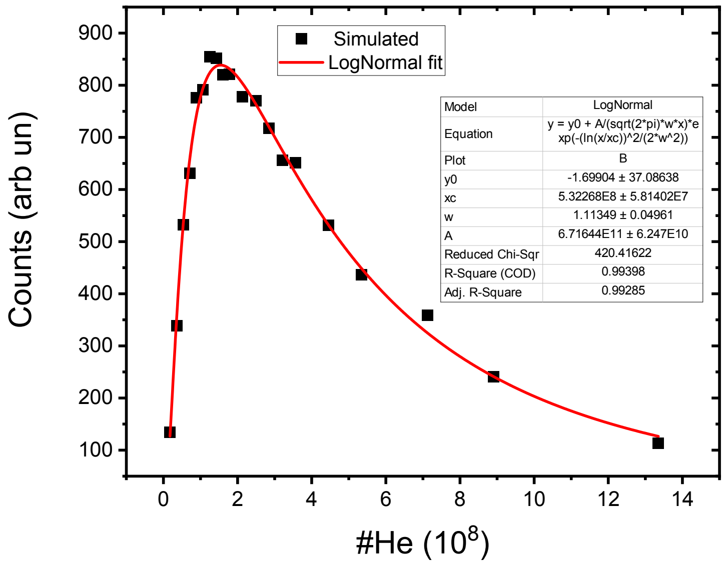

Figure S5. Simulated ion signal for the log-normal distribution of Figure 2 using the conversion expression (2). Note that the adjusted center positions are not consistent.

Applying expression 5 to the signal of Figure S5 we obtain the distribution depicted in Figure S6.

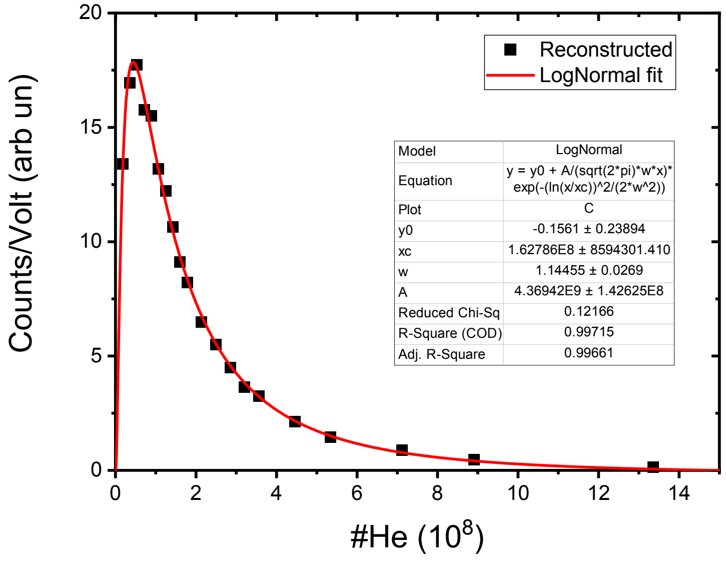

Figure S6. Reconstructed mass distribution of ions transmitted through the energy analyzer using the distribution function of Figure S2 and expressions (2) and (5).

It is clear that within the limitations of statistical noise the distributions obtained in Figure S2 and S6 are in excellent agreement.

B) Experimental Results

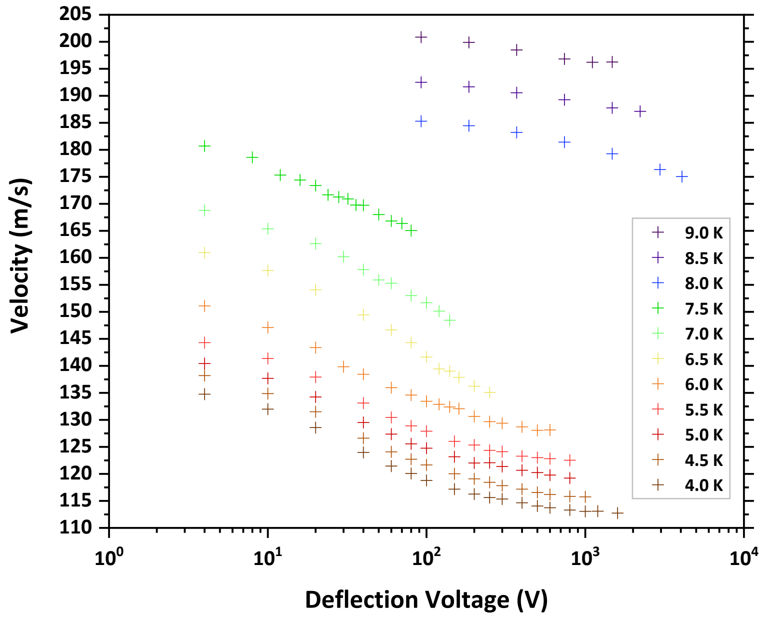

Figure S7. Temperature and deflection voltage dependent droplet velocities. Temperatures 4 to 7.5 K studied with the 5 m radius analyzer, 8 to 9 K studied with the 7 cm radius analyzer.

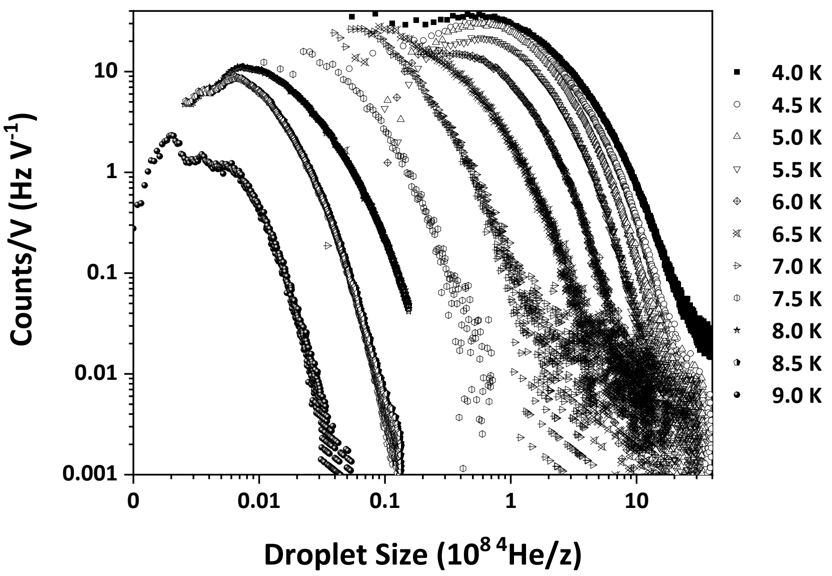

Figure S8. Experimental count rates at the detector as a function of number of Helium atoms per charge for all the investigated temperatures in our study. Temperatures 4 to 7.5 K studied with the 5 m radius analyzer, 8 to 9 K studied with the 7 cm radius analyzer.

The experimental points of Figure S8 were fitted with the expression

$$f(N) = f_0 + \frac{A}{N\, w \sqrt{2\,\pi}} \exp\left(-\frac{1}{2}\left(\frac{\ln\left(\frac{N}{\overline{N}}\right)}{w}\right)^2 \right) \#(6)$$

where $\overline{N}$ is the average droplet size per unit charge, w is the width of the distribution and f_0 and A are free parameters related to noise floor and detection efficiency respectively.

**Temperature (K)**	**Energy analyzer (He/z)**	**Henne [2] (He/z)**	**Samelin [3] (He/z)**	**Vilesov [4] (He)**
4.0	$(1.732\pm0.005)\times10^{8}$			
4.5	$(1.65\pm0.02)\times10^{8}$			
5.0	$(1.316\pm0.001)\times10^{8}$			
5.4				1.68×10^{10}
5.5	$(1.145\pm0.003)\times10^{8}$			
5.7				1.43×10^{9}
6.0	$(7.53\pm0.06)\times10^{7}$			3.03×10^{8}
6.5	$(2.74\pm0.03)\times10^{7}$	3.50×10^{7}		5.90×10^{7}
7.0	$(1.070\pm0.009)\times10^{7}$	2.20×10^{7}		9.64×10^{6}
7.5	$(4.39\pm0.09)\times10^{6}$			
8.0	$(1.65\pm0.04)\times10^{6}$	9.00×10^{6}	5.60×10^{6}	5.31×10^{6}
8.0	$(1.676\pm0.005)\times10^{6}$			
8.5	$(9.85\pm0.02)\times10^{5}$			
9.0	$(5.83\pm0.03)\times10^{5}$	1.80×10^{6}	1.20×10^{6}	1.82×10^{6}
9.5		1.40×10^{5}	1.50×10^{6}	3.25×10^{5}

Table S1. Mean anionic droplet sizes fitted to the data of Figure S8, in comparison with reported literature values. All reported values were obtained at 20 bar expansion pressure. Error bars refer to uncertainties in the fitting procedure. Values between 4 and 8 K were obtained with our 5 m radius analyzer, while the values between 8 and 9.5K were obtained with the 7 cm radius analyzer. Note that the 8 K point is repeated once for each setup.

**Temperature (K)**	**Distribution width**
4.0	1.116 ± 0.002
4.5	1.104 ± 0.006
5.0	0.901 ± 0.002

5.5	0.877 ± 0.002
6.0	0.875 ± 0.006
6.5	0.895 ± 0.009
7.0	0.676 ± 0.008
7.5	0.79 ± 0.02
8.0	0.56 ± 0.02
8.0	0.838 ± 0.002
8.5	0.691 ± 0.002
9.0	0.679 ± 0.008

Table S2. Width of the distributions fitted to the data of Figure S8. Error bars refer to uncertainties in the fitting procedure. Values between 4 and 8 K were obtained with our 5 m radius analyzer, while the values between 8 and 9.5K were obtained with the 7 cm radius analyzer. Note that the 8 K point is repeated once for each setup.

C) Nozzle Image

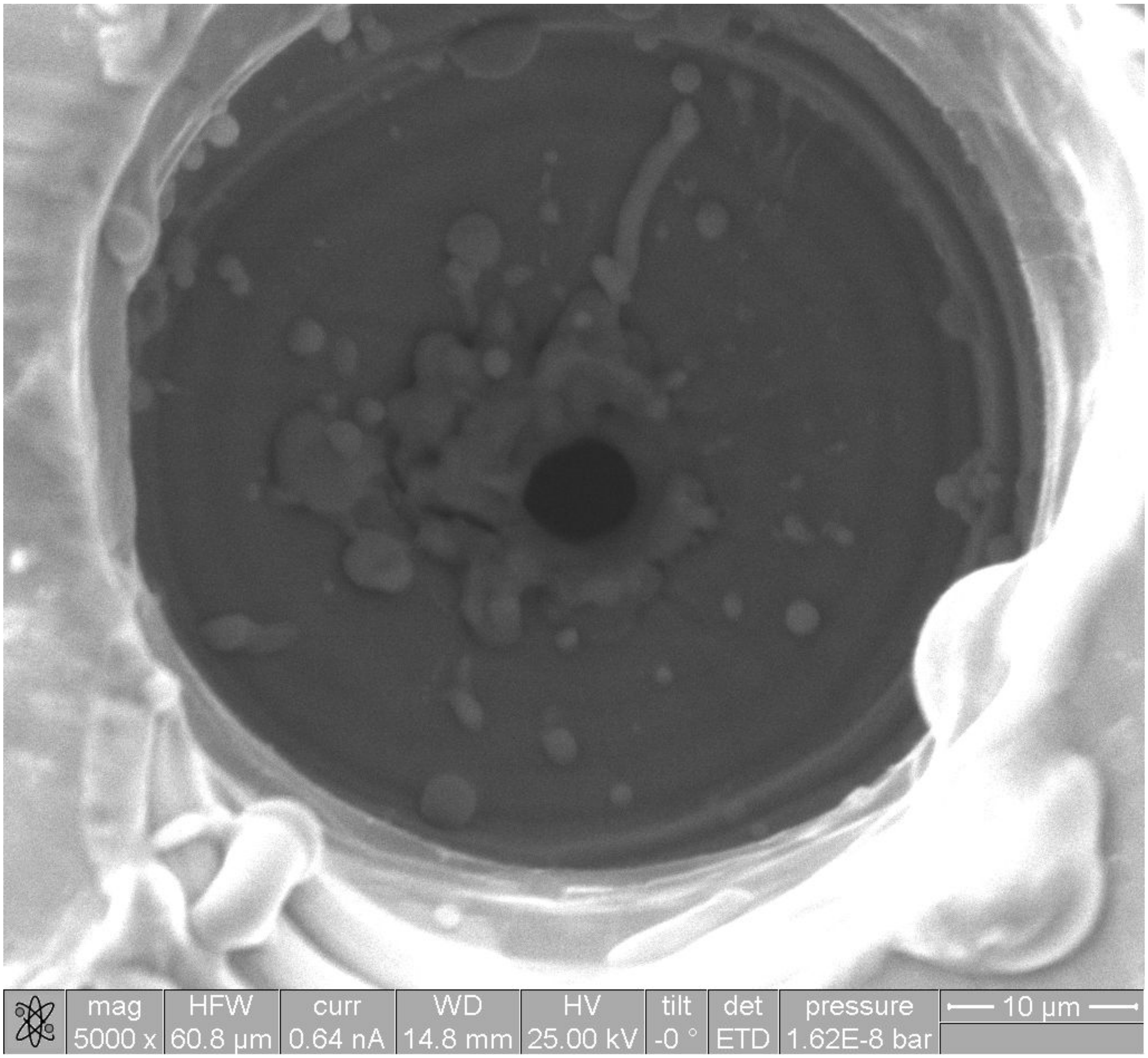

Figure S9. Scanning Electron Microscope image of the laser drilled SST nozzle prior to installation. The image shows the exit side of the nozzle with the 5.4 µm nozzle opening in the 50 µm exit tunnel. Image taken by David Stock (Unit of Material Technology Innsbruck, University Innsbruck) recorded with a FEI Quanta 200 3D SEM.

REFERENCES

1. Fárník, M.; Henne, U.; Samelin, B.; Toennies, J. P. Comparison between Positive and Negative Charging of Helium Droplets. *Z. Phys. D* **1997,** *40*, 93-98.
2. Henne, U. Available as Bericht 5/1996. Ph.D. dissertation, Max-Planck-Institut für Strömungsforschung, Göttingen, Germany, 1996.

3. Samelin, B. Available as Bericht 16/1998. Ph.D. dissertation, Max-Planck-Institut für Strömungsforschung, Göttingen, Germany, 1998.
4. Gomez, L. F.; Loginov, E.; Sliter, R.; Vilesov, A. F. Sizes of Large He Droplets. *J. Chem. Phys.* **2011,** *135*, 154201.
